# Inhibition of skin carcinogenesis by suppression of NF-κB dependent ITGAV and TIMP-1 expression in IL-32γ overexpressed condition

**DOI:** 10.1186/s13046-018-0943-8

**Published:** 2018-11-28

**Authors:** Yong Sun Lee, Chung Hee Lee, Jun Tae Bae, Kyung Tak Nam, Dae Bong Moon, Ok Kyung Hwang, Jeong Soon Choi, Tae Hoon Kim, Hyoung Ok Jun, Young Suk Jung, Dae Yeon Hwang, Sang-Bae Han, Do Young Yoon, Jin Tae Hong

**Affiliations:** 1 College of Pharmacy and Medical Research Center, Chungbuk National University, Osongsaengmyeong 1-ro, Osong-eup, Heungdeok-gu, Cheongju, Chungbuk 28160 Republic of Korea; 2 Hanbul Co, Ltd. R&D center, 634 Eon Ju-Ro, Gangnam-gu, Seoul, Republic of Korea; 3 Department of Biomaterial Science, Pusan National University, Miryang, Kyungnam 50463 Republic of Korea; 4 Department of Bioscience and Biotechnology, Bio/Molecular Informatics Center, Konkuk University, Gwangjin-gu, Seoul, 05029 Republic of Korea

**Keywords:** IL-32γ, TIMP-1, ITGAV, NF-κB, Skin tumor development

## Abstract

**Background:**

Interleukin-32 (IL-32) has been associated with various diseases. Previous studies have shown that IL-32 inhibited the development of several tumors. However, the role of IL-32γ, an isotype of IL-32, in skin carcinogenesis remains unknown.

**Methods:**

We compared 7,12-Dimethylbenz[a]anthracene/12-O-Tetradecanoylphorbol-13-acetate (DMBA/TPA)-induced skin carcinogenesis in wild type (WT) and IL-32γ-overexpressing mice to evaluate the role of IL-32γ. We also analyzed cancer stemness and NF-κB signaling in skin cancer cell lines with or without IL-32γ expression by western blotting, quantitative real-time PCR and immunohistochemistry analysis.

**Results:**

Carcinogen-induced tumor incidence in IL-32γ mice was significantly reduced in comparison to that in WT mice. Infiltration of inflammatory cells and the expression levels of pro-inflammatory mediators were decreased in the skin tumor tissues of IL-32γ mice compared with WT mice. Using a genome-wide association study analysis, we found that IL-32 was associated with integrin αV (ITGAV) and tissue inhibitor of metalloproteinase-1 (TIMP-1), which are critical factor for skin carcinogenesis. Reduced expression of ITGAV and TIMP-1 were identified in DMBA/TPA-induced skin tissues of IL-32γ mice compared to that in WT mice. NF-κB activity was also reduced in DMBA/TPA-induced skin tissues of IL-32γ mice. IL-32γ decreased cancer cell sphere formation and expression of stem cell markers, and increased chemotherapy-induced cancer cell death. IL-32γ also downregulated expression of ITGAV and TIMP-1, accompanied with the inhibition of NF-κB activity. In addition, IL-32γ expression with NF-κB inhibitor treatment further reduced skin inflammation, epidermal hyperplasia, and cancer cell sphere formation and downregulated expression levels of ITGAV and TIMP-1.

**Conclusions:**

These findings indicated that IL-32γ suppressed skin carcinogenesis through the inhibition of both stemness and the inflammatory tumor microenvironment by the downregulation of TIMP-1 and ITGAV via inactivation of NF-κB signaling.

**Electronic supplementary material:**

The online version of this article (10.1186/s13046-018-0943-8) contains supplementary material, which is available to authorized users.

## Introduction

Interleukin-32 (IL-32), formerly known as natural killer cell transcript 4, has six splice variants, namely IL-32α, −32β, −32γ, −32δ, −32ε, and -32ζ, all of which demonstrate distinct functional differences [1, 2]. IL-32 consists of eight small exons and the second exon contains an ATG start codon. IL-32γ is the longest isoform. IL-32α lacks two of the splicing variant regions found in IL-32γ (spliced between exons 3 and 4, and exons 7 and 8). IL-32β lacks the second exon, is spliced form between exons 3 and 4 of IL-32γ. IL-32δ lacks the second exon, which results in a shift in the ATG codon in the third exon. IL-32ε lacks exons 3 and 4 of IL-32γ. IL-32ζ lacks the second and third exons, and the ATG codon of IL-32ζ is shifted in the fourth exons. It is well known that IL-32 plays significant pathophysiological roles in the development of several inflammatory diseases, such as arthritis, psoriasis, ulcerative colitis, Crohn’s disease, and chronic obstructive pulmonary disease [3–6], since it alters the release of pro and anti-inflammatory cytokines such as tumor necrosis factor-alpha (TNF-α), IL-1β, IL-6, and IL-10 [5, 7, 8]. It has also been reported that IL-32 was critically associated with the development of several cancers [9, 10]. Our recent studies have revealed that IL-32α induced TNFR1-mediated cell death signaling to inhibit tumor development in carcinogen-induced colon tumors, while IL-32β and IL-32γ suppressed tumor growth through the inactivation of STAT3 and NF-κB signaling in xenograft and allograft animal [10–12]. In addition, IL-32γ and IL-32θ inhibit stemness and epithelial-mesenchymal transition (EMT) in colon cancer [13]. It was also reported that IL-32α induces cell apoptosis in human melanoma cells and inhibits EMT in pancreatic cancer cells [14, 15]. However, IL-32α induces human melanoma migration, while IL-32γ increases gastric cancer migration and invasion [16, 17]. However, the role of IL-32γ in carcinogen-induced skin tumor growth and its mechanisms of action have not yet been reported.

Skin cancer is the most common cancer and its incidence is constantly increasing every year [18–20]. During the past few decades, the incidence of skin cancer has significantly increased in both the elderly and adolescents due to increases in chemical carcinogen exposure, ultraviolet exposure, tanning behavior of youth, and outdoor activities [19]. Several reports have demonstrated that chronic inflammation promoted epidermal cell transformation and malignant progression by enhancing the release of cytokines and chemokines and by infiltration of inflammatory cells [21, 22]. Various inflammatory cytokines, including TNF-α, IL-1, IL-6, IL-17, IL-21, and IL-23, promoted skin cancer development [21, 23]. It was also noteworthy that inflammatory cytokines were positively related with cancer stemness. IL-1β was shown to increase cancer sphere formation and stem cell marker expressions in colon cancer [24], while IL-6, IL-8, CCL2 and transforming growth factor-β promoted breast cancer stemness [25]. Tumor-associated macrophages and T_h_2 cell-produced tumor necrosis factor (TNF)-α have been reported to promote cancer stem cell (CSC) plasticity [26]. CSCs, a rare population of tumor cells, are able to preserve tumor heterogeneity with their self-renewal capacity and clonal long-term repopulation potential [26]. Growing evidence has shown that CSCs play a critical role in tumor initiation in many cancer types, including skin cancer [27, 28]. The molecular mechanisms between tumor initiation and CSCs have been poorly characterized in chemical-induced skin carcinogenesis model. However, previous studies have revealed that skin stem cells or CSCs obtained from DMBA/TPA-induced skin tumors contribute to initiate tumor development [29, 30]. These results implicated that skin stem cells or CSCs may be important for tumor initiation in chemical-induced skin carcinogenesis model. Mice lacking Sox2, a core stem cell gene, showed delayed DMBA/TPA-induced skin tumor initiation and reduced tumor proliferation and stemness [28, 31]. Expression of Nanog, another stem cell gene, promoted skin squamous cell carcinoma formation [32].

Integrin αv (ITGAV) heterodimers are known to promote or suppress cancer development in epithelial tissues. In the mouse skin, ITGAV cooperates with p53 to transiently promote initial skin cancer development, but ultimately results in decreased tumor growth [33]. Knockout of ITGAV in mice seemed to promote skin cancer development [34]. ITGAV knockdown and ITGAV antagonist treatment reduced cell migration, stemness, and EMT in prostate and bladder cancer [35–37]. It has also been reported that ITGAV positive colon cancer cells showed increased cancer stemness and chemoresistance [38]. TIMP-1 is a member of the family of matrix metalloproteinase inhibitors, which contains four members (TIMP-1, TIMP-2, TIMP-3, and TIMP-4) [39]. Growing evidence has demonstrated that TIMP-1 expression is related to skin cancer progression. TIMP-1-overexpressing melanoma cells showed elevated anchorage-independent growth in soft agar and increased tumorigenesis and lung metastasis in vivo [40]. The assembly of TIMP-1, CD63, and β1-integrins at the cell surface of melanoma cells was involved in the acquisition of an *anoikis*-resistant phenotype [41]. Suppression of TIMP-1 secretion inhibited anchorage-independent growth of hepatic adenocarcinoma cells [42]. With the data analysis using a genome-wide association study (GWAS), we found that IL-32 was closely related to many cancers (Additional file 1: Figure S1), and further analysis showed that IL-32 was closely associated with several genes including ITGAV and TIMP-1 (Additional file 2: Figure S2A and S2B), which have been implicated in skin tumor development.

Nuclear transcription factor-κB (NF-κB) has been known to be an important factor in inflammation and cancer. In skin cancer, enhanced NF-κB activity leads to hyperproliferation and dysplasia of mouse epidermis. Epidermal keratinocyte-specific deletion of p65 protects DMBA/TPA-induced skin carcinogenesis [43]. NF-κB is activated by cytokines in tumors and tumor environments. Proinflammatory cytokines, IL-1α, IL-6 and IL-8, activate the NF-κB signaling during tumor growth and metastasis. However, NF-κB activity was inhibited by IL-10, which is an important cytokine for anti-tumor immunity and inhibits melanoma growth and metastasis [44–46]. We have previously found that IL-32γ inhibited colon cancer development via inactivation of NF-κB signaling [10]. IL-32β also prevented tumor growth in colon and prostate cancers through the downregulation of NF-κB [47]. NF-κB activation induced tumor development by increasing TIMP-1 expression in KrasG12D lung cancer model [48]. In triple-negative breast cancer cells, NF-κB activation promoted cell proliferation via TIMP-1 expression [49]. ITGAV induced colorectal cancer cell invasion mediated by activation of NF-κB signaling [50]. The NF-κB signal is closely related to cancer stemness. NF-κB activation led to induce expression of stem cell genes and CSC markers including CD133 and Sox2. It has also been reported that NF-κB controls the expression of cytokines, specifically IL-6, which contributed to the survival and self-renewal of CSCs [51].

Thus, in the present study, we investigated whether overexpression of IL-32γ could contribute to skin cancer development through changes in cancer stemness and the inflammatory tumor microenvironment via mediation of these network genes.

## Materials and methods

### Animals

Wild-type (WT, C57BL/6 J) mice were purchased from DBL (Eumsung, Korea). The IL-32γ mice were obtained descried in the previous study [10]. In brief, a 705-base pair fragment of the hIL-32γ gene was subcloned into the EcoRI sites of the pCAGGS expression vector. IL-32γ insertion was confirmed by amplification of genomic DNA isolated from the transgenic mice tails using Super Taq PLUS Pre-mix (RexGeneBioTech, Seoul, Korea) and the following specific primer set: sense, 5′-GAAGGTCCTCTCTGATGACA-3′ and antisense, 5′-GAAGAGGGACAGCTATGACTG-3′ (nt 2245–2225). Genomic DNA samples were extracted from transgenic mice tails and PCR analysis was performed for IL-32γ gene expression. WT mice do not express IL-32γ. IL-32γ transgenic mice have no overt phenotype compared with WT mice. IL-32γ mice were viable, fertile and have no tissue or organ abnormalities. The mice were housed and bred under specific pathogen free conditions at the Laboratory Animal Research Center of Chungbuk National University, Korea. The mice (*n = 8*) were maintained in a room with a constant temperature of 22 ± 1 °C, relative humidity of 55 ± 10%, and 12-h light/dark cycle, and fed standard rodent chow (Samyang Co., Gapyeong, Korea) and purified tap water ad libitum.

### Carcinogenesis protocols

Eight-week old WT and IL-32γ mice were used. Skin carcinogenesis was performed as previously described [52]. The dorsal skin of the mice was shaved, and the exposed areas were treated with 25 μg DMBA (Sigma, St. Louis, MO, USA) in 200 μL of acetone per mouse. After 1 week, 5 μg TPA (Sigma, St. Louis, MO, USA) in 200 μL of acetone was applied per mouse thrice a week. Mice were evaluated weekly for papilloma development. Mice were euthanized after a 25-week TPA treatment. At the time of sacrifice, the skin was fixed in 4% formalin solution. After fixation, the skin was used for surface tumor number and diameter measurements and then embedded in paraffin. Tumor counts were averaged and statistically analyzed. Tumor diameters were measured using Fisherbrand Traceable digital calipers (Fisher Scientific, Asheville, NC, USA). Then, skin tissues were embedded in paraffin. To induce skin inflammation, single TPA was applied on the shaved mice. The shaved mice were treated with or without 0.1 μmol Bay 11–7082 in 200 μL of acetone per mouse. After 1 h, mice were treated with 10 μg TPA in 200 μL of acetone per mouse. Mice were sacrificed after 24 h.

### Cell culture

A431 and SK-MEL-28 human skin cancer cells were obtained from the Korean Cell Line Bank (Seoul, Korea). A431 cells were grown in RPMI1640 supplemented with 10% fetal bovine serum, 100 U/mL penicillin, and 100 μg/mL streptomycin. SK-Mel-28 cells were grown in MEM supplemented with 10% fetal bovine serum, 1 mM sodium pyruvate, 1× MEM non-essential amino acid, 100 U/mL penicillin, and 100 μg/mL streptomycin. Cells were cultured at 37 °C in 5% CO_2_ humidified air.

### Sphere formation assay

A431 and SK-Mel-28 cells were cultured in stem cell media consisting of DMEM/F12 basal media supplemented with N2 and B27 supplements (Invitrogen), 20 ng/mL human recombinant epidermal growth factor (EGF; PeproTech Inc., Rocky Hill, NJ) and 20 ng/mL basic fibroblastic growth factor (bFGF; PeproTech Inc., Rocky Hill, NJ). Before assay, 2-hydroxyethyl methacrylate (poly-HEMA) solution was prepared by mixing 1.2 g poly-HEMA in 100 ml 95% ethanol and heating at 65 °C. Plates were coated by poly-HEMA solution in a sterile hood for 8 h. Poly-HEMA coated plates were sterilized by UV irradiation for 15 min. For the sphere formation assay, cells were plated at a density of 1 × 10^4^ cells/well in poly-HEMA-coated 12-well plates. After 10 days, number of spheres were counted.

### Transfection

Skin cancer cells were transiently transfected with pcDNA3.1(+)-6xMyc-IL-32γ vector or control vector using the Lipofectamine® 3000 transfection reagent in OPTI-MEM, according to the manufacturer’s specification (Invitrogen, Waltham, MA, USA). For stable cell lines, transfected cells were cultured in growth medium containing 600 μg/ml G418 (Geneticin™; Gibco, Grand Island, NY, USA) for 2 weeks. G418-resistant colonies were selected and expanded. For siRNA transfection, negative control (NC), ITGAV and TIMP-1 siRNA were purchased from Santa Cruz Bio (Dallas, TX, USA).

### Chemotherapy resistance assay

Cells were seeded in 96-well plates at 2.5 × 10^3^ cells/well. After 24 h, the cells were treated with 5-fluorouracil (5-FU; 100 μg/ml). The cells were incubated for 24 h. Then, cell viability was measured by performing a thiazolyl blue tetrazolium bromide (MTT) assay. For MTT assay, 10% vol/vol of 5 mg/ml MTT (Sigma, St. Louis, MO, USA) diluted in PBS was added to cancer cell cultures. After 2 h of incubation, the medium was aspirated, and DMSO was added. Absorbance was measured at 570 nm. The data were normalized to their respective controls and are presented as a bar graph.

### Quantitative real-time PCR

Total RNA of skin tissues from WT mice and IL-32γ mice was extracted by Ribo^EX^ RNA Extraction Kit (Gene All biotechnology, Seoul, Korea) and cDNA was synthesized using High Capacity RNA-to-cDNA kit (Applied Biosystems, Foster City, CA, USA). Quantitative real-time PCR was performed using QuantiNova SYBR^®^ Green RT-PCR kit (Qiagen, Hilden, Germany) with  specific primers in a StepOnePlus™ Real-Time PCR System (Applied Biosystems, Foster City, CA, USA) (Additional file 3: Table S1). Thermocycling conditions consisted of an initial denaturation of 20 s at 95.0 °C, followed by 40 cycles of 95.0 °C for 30 s and 60.0 °C for 30 s. The values obtained for the target gene expression were normalized to GAPDH or β-actin and quantified relative to the expression in control samples.

### Immunohistochemistry

Human skin tumor tissue microarrays were purchased from US Biomax (SK801b; Rockville, MD, USA). Paraffin-embedded human and mouse tumor tissue sections were blocked for 60 min with 2% normal horse or goat serum diluted in PBS. The sections were then blotted and incubated with specific primary antibodies in blocking serum for overnight at 4 °C. The next day, the slides were washed three times for 5 min each in PBS and incubated in biotinylated anti-mouse, rabbit, rat or goat antibody for 1 h. The slides were washed in PBS, followed by formation of the avidin-biotin-peroxidase complex (ABC, Vector Laboratories, Inc., Burlingame, CA, USA). The slides were washed, and the peroxidase reaction developed with diaminobenzidine and peroxide, then counterstained with hematoxylin, mounted in Cytoseal XYL (Thermo Fisher Scientific, Waltham, MA, USA), and evaluated using a light microscope (× 200, Olympus, Tokyo, Japan). Specific primary antibodies were purchased from Santa Cruz Bio (PCNA, F4/80 and p50; Dallas, TX, USA), eBioscience (Ly6G and CD11b; Thermo Fisher Scientific, Waltham, MA, USA), Abnova (CD133; Taipei, Taiwan), Novus Biologicals (TIMP-1; Littleton, CO, USA) and Abcam (IL-32, CD44, ITGAV and p65; Cambridge, MA, USA).

### Prostaglandin E2 quantification

Serum level of mouse prostaglandin E2 were measured by enzyme-linked immunosorbent assay (ELISA) kits provided by R&D systems (Minneapolis, MN, USA) according to the manufacturer’s protocol.

### Western blot analysis

Skin tissues from WT mice and IL-32γ mice were lysed by Pro-prep protein extraction buffer (iNtRON, Sungnam, Korea) and the total protein concentration was determined using the Bradford reagent (Bio-Rad, Hercules, CA, USA). Nuclear extraction was performed using nuclear extraction kit (Abcam, Cambridge, MA, USA). The membranes were immunoblotted with specific primary antibodies. The intensity of the bands was measured using the Fusion FX 7 image acquisition system (Vilber Lourmat, Eberhardzell, Germany). Specific primary antibodies were purchased from Santa Cruz Bio (p-IKKα/β, IKKα/β, p-JNK, JNK, p-ERK, p-p38, p38, p-STAT3, STAT3, p50, Histone H1, Cyclin D1, CDK4, Bax, Bcl-2, MMP-2, MMP-9 and β-actin; Dallas, TX, USA), Cell Signaling Technology (Myc-tag and ERK; Trask Lane, Danvers, MA, USA), Abnova (CD133; Taipei, Taiwan), Novus Biologicals (TIMP-1, iNOS and COX-2; Littleton, CO, USA) and Abcam (CD44, ITGAV, S100A8 and p65; Cambridge, MA, USA). β-actin and histone H1 was used as a loading control.

### Electrophoretic mobility shift analysis

DNA binding activity of NF-κB was determined using an electrophoretic mobility shift assay (EMSA). Gel-shift assays were performed according to the manufacturer’s recommendations (Promega, Madison, WI).

### Gene and gene-disease network analyses

The gene network of IL-32 was analyzed using the web-based analysis tool GeneMANIA (www.genemania.org) based on the publicly available biological datasets (gene-gene interactions based on attributions: co-expression, co-localization, genetic interactions, pathway, physical interactions, predicted interactions and shared protein domains). The gene network is automatically analyzed by gene-ontology base weighting methods. The gene-disease network of IL-32 was analyzed using the DiseaseConnect (http://disease-connect.org) resource. This web server analyzed gene-disease network based on various sources, such as DEG, GWAS, OMIM, GeneRIF and GeneWays. The strength of the connections between an input gene and diseases is quantified as the *p*-value of a hypergeometric enrichment test in the number of shared genes.

### Data analysis

The data were analyzed using the GraphPad Prism software ver 4.03 (San Diego, CA, USA). Data are presented as mean ± S.E.M. The differences in all data were assessed by one-way analysis of variance (ANOVA). When the *P* value in the student’s *t*-test indicated statistical significance, the differences were assessed by the Dunnett’s test. A value of *p* < 0.05 was considered to be statistically significant.

## Results

### IL-32γ inhibits skin tumor development

**Fig. 1:**
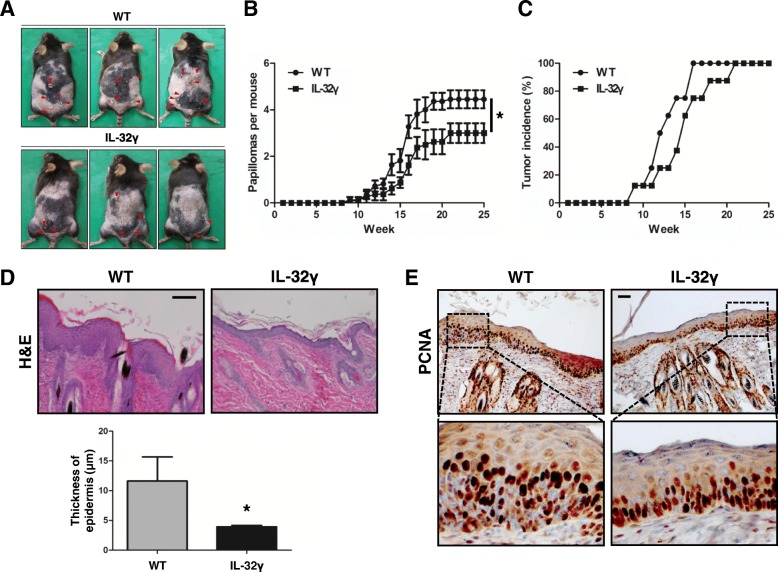
Effect of IL-32γ on skin tumor development. **a** Representative images of WT and IL-32γ mice with skin papillomas. **b** Average number of papillomas per mouse in WT and IL-32γ mice following TPA treatment. *n = 8*. *p < 0.05. **c** Tumor incidence, showing the percentage of tumor-bearing mice at time course. *n = 8*. **d** hematoxylin and eosin (H&E) staining of skin sections and epidermal thickness in WT and IL-32γ mice. *n = 8*. *p < 0.05. **e** PCNA staining of skin sections in WT and IL-32γ mice. Scale bar, 10 μm

First, we investigated the effect of IL-32γ on skin tumor development. Skin carcinogenesis was initiated by a single treatment with 7,12-dimethylbenz[a]anthracene (DMBA), and then promoted by 12-O-tetradecanoylphorbol-13-acetate (TPA) treatments for 25 weeks. IL-32γ mice showed a significantly reduced number of skin papillomas (Fig. 1a). The number of papillomas was 2.75 ± 1.03 per IL-32γ mouse compared to 5.125 ± 0.99 per WT control (Fig. 1b). Tumor development was delayed in IL-32γ mice compared to in WT mice (Fig. 1c). Histological analysis of skin sections from IL-32γ mice showed reduced epidermal thickening and hyperplasia compared to WT mice (Fig. 1d). The number of PCNA-positive cells was smaller in IL-32γ mice than in the WT controls (Fig. 1e). Additional western blot analysis showed that the expression levels of proliferation (PCNA, CDK4, and cyclin D1), metastasis (MMP-2 and MMP-9) and inflammation (COX-2 and iNOS) markers were decreased in skin tissues of IL-32γ mice compared to those of WT mice (Additional file 4: Figure S3). It has been previously demonstrated that DMBA/TPA induced skin carcinogenesis through MAPK and STAT3 signaling [53, 54]. We identified the activation of JNK, ERK, p38, and STAT3 in skin tissues from WT and IL-32γ mice. Phosphorylation of JNK, p38, and STAT3 was reduced, but the phosphorylation status of ERK did not change in skin tissues from IL-32γ mice compared to that from WT mice (Additional file 4: Figure S3).

### IL-32γ inhibits local skin inflammation and affected inflammatory cell number

**Fig. 2:**
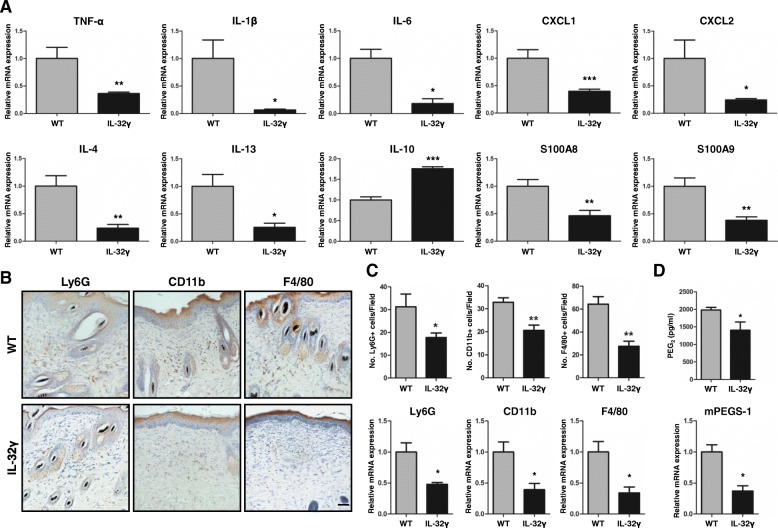
IL-32γ effects on DMBA/TPA-induced local inflammation and inflammatory cell infiltration. WT and IL-32γ mice were treated with DMBA/TPA for 25 weeks. **a** Real-time PCR analysis of different inflammatory mediators, TNF-α, IL-1β, IL-6, IL-4, IL-10, IL-13, CXCL1, CXCL2, S100A8 and S100A9, on mRNA isolated from skin tissue extracts. *n = 5.* *p < 0.05; **p < 0.01; ***p < 0.001. **b** Representative immunohistochemistry images showing Ly6G+ (granulocytes), CD11b + (monocytes/phagocytes) and F4/80+ (macrophages) cells in the skin sections of WT and IL-32γ mice. Ly6G, CD11b and F4/80 stainings were quantified by counting the number of positive cells in the field. Scale bar, 10 μm. *n = 5*. **c** Real-time PCR analysis of mRNA expression of Ly6G, CD11b and F4/80. *n = 6*. *p < 0.05; **p < 0.01. **d** Production of PGE_2_ in the skin tissues measured by ELISA and mRNA expression of mPGES-1 measured by real-time PCR. *n = 6*. *p < 0.05

Many studies revealed that local inflammation and infiltration of immune cells was correlated with skin cancer development. To investigate whether the reduced skin inflammation and infiltration of immune cells into skin were associated with IL-32γ mice with reduced skin carcinogenesis, we investigated the level of Th1/M1 pro-inflammatory cytokines, Th2/M2 anti-inflammatory cytokines and chemokines in DMBA/TPA-induced skin tissues. We found that mRNA levels of Th1/M1 pro-inflammatory mediators, such as IL-1β, IL-6 and TNF-α, were decreased in IL-32γ mice compared to that in WT mice (Fig. 2a). However, Th2/M2 anti-inflammatory cytokines, including IL-4 and IL-13, were reduced, but IL-10 was increased in IL-32γ mice compared to that in WT mice (Fig. 2a). S100A8, S100A9, CXCL1 and CXCL2, chemoattractant chemokines which are important factors for recruiting inflammatory cells, were also decreased in the skin tissue from IL-32γ mice compared to that from WT mice (Fig. 2a). Moreover, we found a reduced recruitment of Ly6G+ granulocytes, CD11b + monocytes/phagocytes and F4/80+ macrophages in the skin tissues of IL-32γ mice compared to that in WT mice (Fig. 2b). In line with these findings, IL-32γ mice had lower mRNA expression levels of Ly6G, CD11b, and F4/80 compared to WT mice (Fig. 2c). In addition, the level of the inflammatory mediator prostaglandin E_2_ (PGE_2_), and mRNA expression of PGE_2_ synthase mPGES-1 were also significantly reduced in skin tissues from IL-32γ mice compared to those from WT mice (Fig. 2d).

### IL-32γ suppresses skin cancer stemness

**Fig. 3:**
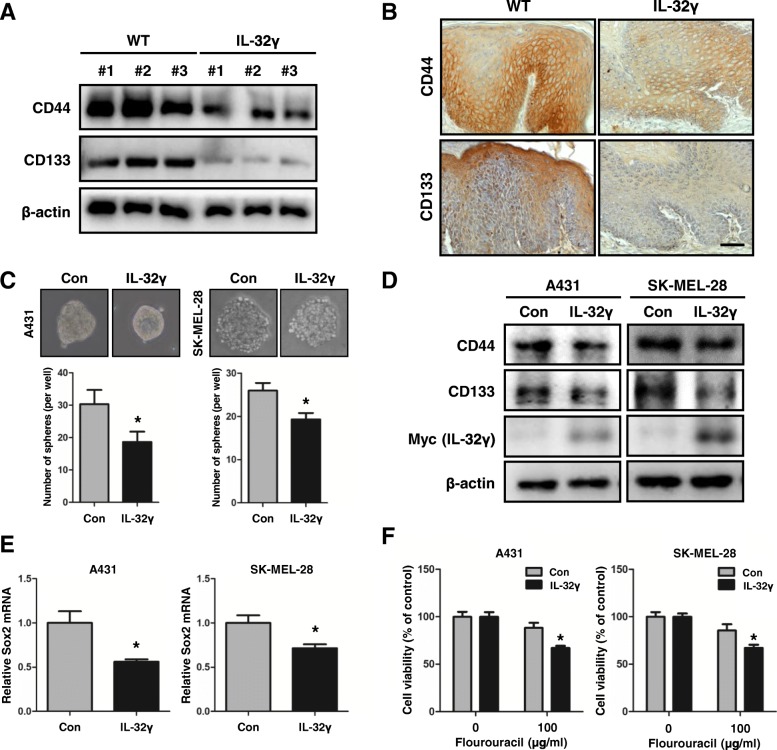
IL-32γ suppresses skin cancer stemness. **a** and **b** Effect of IL-32γ on the expression of CD44 and CD133 in skin tumor by western blotting (**a**) and immunohistochemical analysis (**b**). Scale bar, 10 μm. *n = 4*. **c** Effects of IL-32γ on skin cancer sphere formation. Control and IL-32γ-overexpressing A431 and SK-Mel-28 cells were subject to sphere assay for 10 days. Representative images (top) of skin cancer spheres are shown. *n = 3.* *p < 0.05. **d** Expression of CD44 and CD133 were detected in A431 and SK-Mel-28 skin CSCs by western blotting. **e** Expression of Sox2 was analyzed by real-time PCR. *n = 3*. *p < 0.05. **f** Effects of IL-32γ on skin cancer chemotherapy. Skin cancer cells, A431 and SK-Mel-28, were cultured with 5-FU for 24 h. The cell viability was determined by MTT assay. *n = 3*. *p < 0.05

Next, we investigated whether IL-32γ inhibited skin cancer cell growth. Unexpectedly, no significant differences were observed for the in vitro proliferation rates between the control and IL-32γ-overexpressing cells in HaCaT, A431 and SK-Mel-28 cell lines (Additional file 5: Figure S4). During cancer progression, CSCs are involved in tumor initiation and progression [55]. CSCs, known as tumor-initiating cells, have the potential for self-renewal, clonal tumor initiation, and clonal long-term repopulation. Thus, CSCs can initiate and sustain aggressive tumor growth [26]. We questioned whether IL-32γ inhibited cancer stemness, and thus IL-32γ reduced tumor development. First, we investigated whether expression of CSC markers was inhibited in DMBA/TPA-induced skin tissues of IL-32γ mice. As expected, the expression levels of CD44 and CD133 were much lower in the skin tissues of IL-32γ mice compared to WT mice as evaluated by western blotting and immunohistochemistry (Fig. 3a and b). It was also found that the number and size of spheroid bodies was reduced in IL-32γ-overexpressing A431 and SK-MEL-28 cells compared with those in the control A431 and SK-Mel-28 cells (Fig. 3c). In accordance with reduced stemness, the expression of CSC markers, CD44 and CD133, were decreased in IL-32γ overexpressing skin cancer cells (Fig. 3d). In addition, the mRNA expression of Sox2 was also reduced in IL-32γ overexpressing cells (Fig. 3e). CSCs may play a critical role in cancer chemoresistance by enhanced aldehyde dehydrogenase (ALDH) activity and DNA damage response [56]. Moreover, previous studies have revealed that CD44- or CD133-positive CSCs were highly resistant to chemotherapy [57]. We examined whether IL-32γ affected cancer chemotherapy resistance. A cell viability assay using 5-fluorouracil (5-FU) showed that IL-32γ induced the effect of 5-FU treatments by increasing skin cancer cell death in A431 and SK-Mel-28 cells (Fig. 3f). Our findings suggest that IL-32γ inhibited CSC properties in skin cancer cells.

### Expression levels of ITGAV and TIMP-1 are associated with the inhibition of IL-32γ-induced cancer stemness

**Fig. 4:**
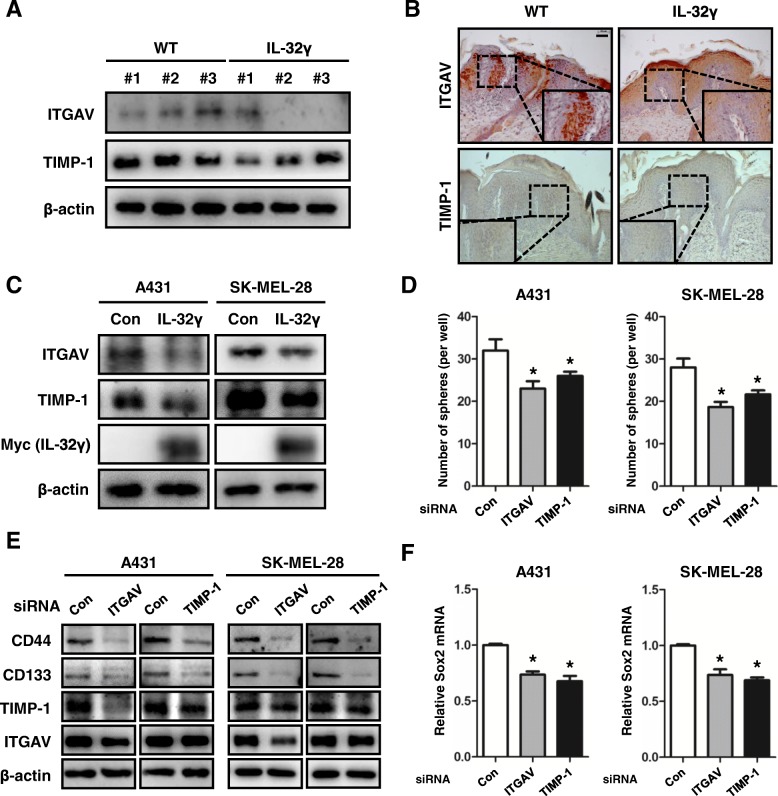
ITGAV and TIMP-1 are associated with inhibition of IL-32γ-induced cancer stemness. **a**, **b** Western blotting (**a**) and immunohistochemical (**b**) analysis of ITGAV and TIMP-1 in DMBA/TPA-induced skin tissues from WT and IL-32γ. Scale bar, 10 μm. *n = 4*. **c** Western blotting analysis of ITGAV and TIMP-1 in control and IL-32γ-overexpressing cells (A431 and SK-Mel-28). **d** Knockdown of ITGAV and TIMP-1 on skin cancer sphere formation. A431 and SK-Mel-28 cells were transfected with ITGAV or TIMP-1 siRNA (20 nM). After 24 h, cells were subject to sphere assay for 10 days. *n = 3*. *p < 0.05. **e**, A431 and SK-Mel-28 cells were transfected with ITGAV or TIMP-1 siRNA. Expression of CD44 and CD133 were detected in ITGAV and TIMP-1 knockdonwed A431 and SK-Mel-28 cells by western blotting. **f** Expression of Sox2 mRNA was analyzed by real-time PCR. *n = 3*. *p < 0.05

Using the GWAS analysis, we found that IL-32 was associated with ITGAV and TIMP-1. Previous studies have shown that the inhibition of ITGAV expression decreased cancer stemness, migration and EMT in bladder carcinoma cells [36]. Elevated TIMP-1 levels contributed to increase colony formation in soft agar [40]. To assess the relationship between IL-32γ and these genes, we evaluated the expression levels of ITGAV and TIMP-1 in IL-32γ-expressing DMBA/TPA-induced skin tissues and skin CSCs. Using western blot analysis, ITGAV and TIMP-1 expression were found to be decreased in DMBA/TPA-induced skin tissues from IL-32γ mice compared to tissues from WT mice (Fig. 4a). Moreover, immunohistochemical analysis showed that the expression levels of these genes were reduced in DMBA/TPA-induced skin tissues of IL-32γ mice (Fig. 4b). We next investigated the expression levels of ITGAV and TIMP-1 in skin CSCs. The expression levels of ITGAV and TIMP-1 were decreased in IL-32γ-overexpressing A431 and SK-Mel-28 CSCs compared to that in control CSCs (Fig. 4c). We further investigated whether ITGAV or TIMP-1 was associated with cancer stemness. We showed that knockdown of ITGAV and TIMP-1 reduced sphere formation in A431 and SK-Mel-28 cells (Fig. 4d). The expression levels of CD44 and CD133 were decreased by knockdown of ITGAV and TIMP-1 in skin cancer cells compared to control cells (Fig. 4e). Also, knockdown of ITGAV downregulated TIMP-1 expression, but not vice versa (Fig. 4e). Additionally, mRNA expression of Sox2 was downregulated in ITGAV and TIMP-1 knockdown A431 and SK-Mel-28 cells (Fig. 4f). These results indicated that IL-32γ suppressed cancer stemness through inhibition of ITGAV-mediated TIMP-1 expression.

### IL-32γ decreases NF-κB activity in tumor tissues and skin cancer stem cells

**Fig. 5:**
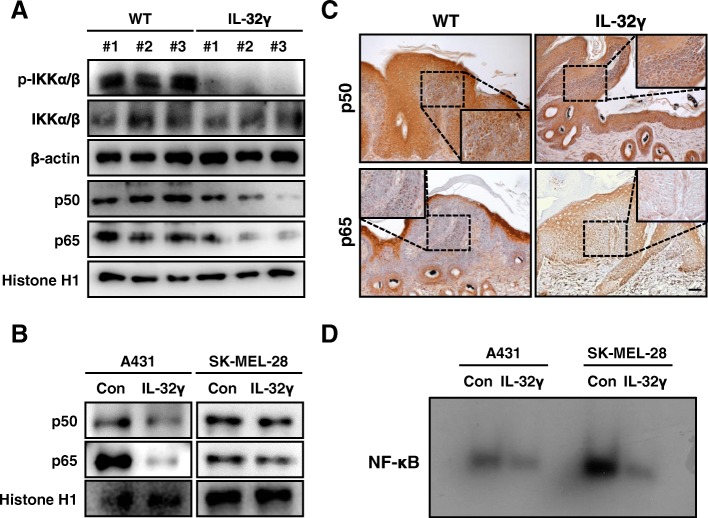
IL-32γ decreases NF-κB activity in tumor tissues and skin CSCs. **a** and **b** Expression of phosphorylated IKKα/β in cytosol extracts and nuclear translocation of p50 and p65 in the nuclear extracts of DMBA/TPA-induced skin tissues or A431 and SK-Mel-28 CSCs were determined by western blotting. **c** Expression of p50 and p65 in DMBA/TPA-induced skin tissues or A431 and SK-Mel-28 CSCs were analyzed by immunohistochemistry. Scale bar, 10 μm. *n = 4.* **d** DNA-binding activity of NF-κB was determined by EMSA in nuclear extracts of A431 and SK-Mel-28 CSCs

NF-κB signaling has a key role in inflammation- and tumor-promoting functions in various tissues [58]. Previous studies revealed that increased expression of p50 was observed during mouse skin carcinogenesis and epidermal knockout of p65 abolished DMBA/TPA-induced skin carcinogenesis [43, 59]. Moreover, NF-κB was closely associated with cancer stemness [51]. To determine whether IL-32γ inhibited the activation of NF-κB in DMBA/TPA-induced skin tissues and CSCs, we conducted western blot and immunohistochemical analyses to examine the translocation of p50 and p65 into the nucleus and phosphorylation of IKKα/β. The translocation of p50 and p65 into the nucleus and phosphorylation of IKKα/β were significantly decreased in the DMBA/TPA-induced skin tissues and IL-32γ-overexpressing skin CSCs (Fig. 5a and b). Immunohistochemical analysis of p50 and p65 confirmed that the nuclear staining intensities for p50 and p65 were decreased in the DMBA/TPA-induced skin tissues of IL-32γ mice compared to controls (Fig. 5c). The DNA-binding activity of NF-κB was lower in IL-32γ-overexpressing skin CSCs compared to controls (Fig. 5d). These data implicated that IL-32γ inactivated NF-κB in skin tissues and skin CSCs, which were associated with the inhibition of skin carcinogenesis.

### Inhibition of NF-κB activity suppresses cancer stemness and skin inflammation

**Fig. 6:**
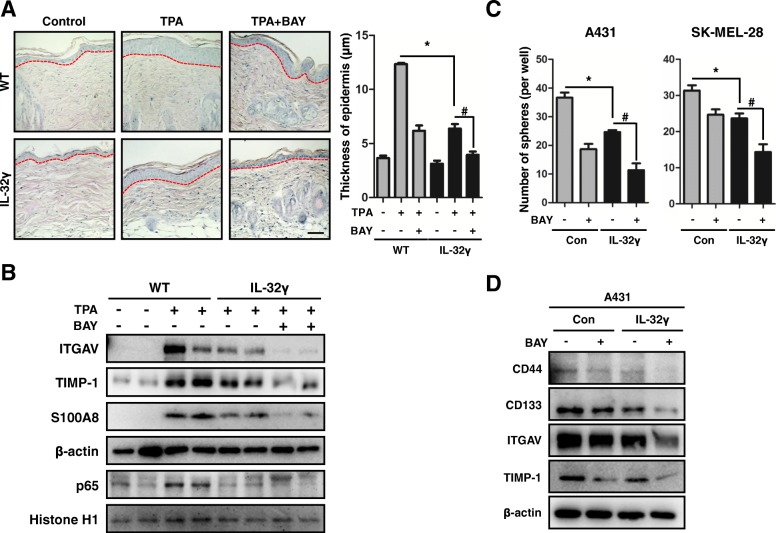
Inhibition of NF-κB activity suppresses cancer stemness and skin inflammation. **a** Effects of BAY on TPA-induced epidermal hyperplasia. WT and IL-32γ mice were topically administrated with BAY and TPA application and then sacrificed after 24 h. Dorsal skin tissues were analyzed by H&E staining. Representative images of skin tissues are shown. The bar graph shows the average epidermal thickness of mice in each group. Scale bar, 10 μm. *n = 3.* *p < 0.05; #*p < 0.05*. **b** Western blotting of ITGAV, TIMP-1 and S100A8 in cytosolic extracts and p65 in nuclear extracts of skin tissues from single TPA-treated with BAY mice are shown. **c** Effects of NF-κB inhibitor Bay 11–7082 (BAY) on skin cancer cell sphere formation. Control and IL-32γ-overexpressing A431 and SK-Mel-28 cells were subject to sphere assay in the presence of Bay (5 μM) for 10 days. **d** Expression of CD44, CD133, ITGAV and TIMP-1 in A431 CSCs. Control and IL-32γ-overexpressing A431 CSCs were treated with BAY (5 μM) for 24 h. Protein levels of CD44, CD133, ITGAV and TIMP-1 were detected by western blotting

We showed that NF-κB signaling was inactivated in skin tissues and skin CSCs. Thus, we further examined the role of NF-κB in cancer stemness and skin inflammation after treatment of the NF-κB inhibitor, BAY 11–7082 (BAY). IL-32γ mice treated with BAY showed substantially reduced TPA-induced epidermal thickness (Fig. 6a). The expression levels of ITGAV, TIMP-1, and inflammation marker S100A8 were more decreased in TPA-induced skin tissues from BAY-treated IL-32γ mice (Fig. 6b). Moreover, local inflammation and inflammatory cell infiltration were more reduced in BAY-treated TPA-induced skin tissues from IL-32γ mice compared with that from controls (Additional file 6: Figure S5A and B). The NF-κB inhibitor treatment demonstrated a significant inhibitory effect on skin CSC formation both in the control and IL-32γ-overexpressing CSCs (Fig. 6c). However, the extent of the inhibition was more significant in IL-32γ-overexpressing CSCs (Fig. 6c). Furthermore, we investigated whether NF-κB signaling was responsible for the expression of ITGAV and TIMP-1 in skin CSCs. The expression levels of ITGAV and TIMP-1 were also decreased in BAY-treated skin CSCs compared to controls (Fig. 6d). Moreover, IL-32γ-overexpressing CSCs with BAY treatment reduced ITGAV and TIMP-1 expression more significantly than IL-32γ-overexpressing CSCs (Fig. 6d). The expression levels of CD44 and CD133 were also more significantly reduced in BAY treated IL-32γ-overexpressing CSCs compared to CSCs. (Fig. 6d). These data indicate that the inhibition of NF-κB was implicated in IL-32γ-induced inhibition of skin carcinogenesis by suppression of ITGAV-mediated TIMP-1 dependent stemness and local inflammation.

### IL-32, ITGAV, and p65 expression in skin tumor tissues of patients

**Fig. 7:**
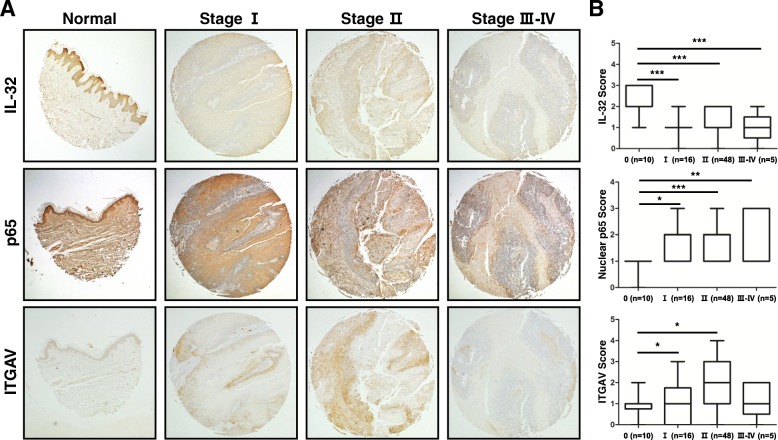
IL-32, ITGAV and p65 expressions in the progression of human skin cancer patients. Tissue microarray analysis showing the expression of IL-32 (top), ITGAV (middle) and p65 (bottom) during skin tumor progression in normal, clinical stage I, II and III-IV. **a** Representative immunohistochemical images of each groups. **b** Bar graphs showing the ratio of IL-32, ITGAV and p65 expressions scored. Tissue microarray contained of 10 samples from normal skin tissues, 16 samples from stage I, 48 samples from stage II and 5 samples from stage III-IV. *p < 0.05

In this study, the expression of IL-32γ resulted in reduced skin tumor development by downregulating ITGAV and TIMP-1 expression through the regulation of NF-κB signaling in IL-32γ mice. We analyzed the expressions levels of IL-32γ, nuclear p65 and ITGAV in patient skin tumor tissues at different stages by immunohistochemical staining analysis. IL-32 expression was reduced in late stage skin tumor tissues (Fig. 7a and b). However, the expression of nuclear translocation of p65 and ITGAV were increased in the late stage skin tumors compared to normal tissues (Fig. 7a and b).

## Discussion

In this study, we found that IL-32γ mice showed an inhibitory effect on carcinogen-induced skin tumor development in comparison with WT mice. This observation extended our previous study, showing anti-tumor effects of IL-32 in melanoma, colon and prostate tumors [10, 47]. However, in the present study we demonstrated that the inhibitory effect of IL-32γ on skin cancer development was associated with preventive effects of IL-32γ on cancer stemness and inflammation.

IL-32, a novel cytokine, is associated with inflammation and cancer development [9]. IL-32 showed a pro-inflammatory effect in various diseases such as arthritis, Crohn’s disease, and inflammatory bowel disease [9]. It has been reported that IL-32α, β, and γ mice showed reduced colon cancer development compared to WT mice due to the inhibition of inflammation, cell cycle arrest or apoptosis in a carcinogen-induced or xenograft model [11, 13]. It has also been reported that colon and prostate tumor developments were reduced in IL-32β mice [12]. However, the role of IL-32γ and the underlying mechanisms in skin cancer development has not yet been reported. In this study, we showed that IL-32γ inhibited skin cancer development by suppressing cancer stemness and inflammation. Unlike previous studies, the present data showed that IL-32γ did not directly inhibit skin cancer cell growth; however, IL-32γ suppressed cancer sphere formation and expression of CSC markers. Chemotherapy resistance is one of the CSC features that are related to cancer recurrence [56]. We also found that IL-32γ diminished chemotherapy resistance of skin cancer cells. In addition, IL-32γ mice had reduced CSC marker protein expression. CSCs have been identified in many tumors, including skin, breast, colon, and prostate cancer, and are known to be involved in tumor development and metastasis [60–63]. The expression of stem cell transcription factors, such as Sox2, Nanog, and Oct4, contributed to tumorigenesis [64]. In our study, IL-32γ suppressed skin cancer sphere formation and the transcriptions of stem cell markers. These findings implied that the reducing effect of IL-32γ on cancer stemness was significant for inhibiting the effect of IL-32γ on skin tumor development.

In chronic inflammation, cytokines, chemokines, and tumor infiltrating inflammatory cells may promote tumor development by favoring the tumor microenvironment [21]. Pro-inflammatory cytokines, such as TNF-α, IL-1β, and IL-6, contribute to the induction of tumor development by enhancing cancer stemness [51, 65]. The pro-inflammatory mediator PGE_2_ promoted CSC expansion [66]. CSCs also elevated secretion of IL-6, RANTES and MCP-1, all of which promoted inflammation, attracted inflammatory cell infiltration and contributed to cancer progression [67, 68]. The production of various inflammatory mediators, such as IL-1β, IL-6, TNF-α, CXCL1, CXCL2, S100A8, S100A9, and PGE_2_, were reduced in skin tissues from DMBA/TPA-treated IL-32γ mice in comparison to WT mice. We also observed that IL-32γ reduced the recruitment of inflammatory cells, Ly6G+ granulocytes, CD11b+ monocytes/phagocytes and F4/80+ macrophages, which were associated with the production of PGE_2_, in skin tissues of DMBA/TPA-treated IL-32γ mice. However, the anti-inflammatory Th2/M2 cytokines IL-4 and IL-13 were not increased in DMBA/TPA-induced skin tissues from IL-32γ mice. IL-4 and IL-13 were key players for activating tumor-associated macrophages and myeloid-derived suppressor cells that promoted tumor development [69]. In addition, IL-4 and IL-13 could increase CSC formation [70, 71]. Protein kinase c (PKC) is an initial molecular target of tumor-promoting agent TPA. IL-32 isoforms have been associated with PKC. IL-32α interacts with PKCε to phosphorylate STAT3 and increased IL-6 expression in TPA-activated THP-1 cells [72]. IL-32β upregulated IL-10 expression by mediating C/EBPα phosphorylation (Serine 21) by PKCδ in TPA-activated U937 cells [73]. IL-32θ suppressed CCL5 expression by interacting with PKCδ and phosphorylates STAT3 (Serine 727) in TPA-activated THP-1 cells [74]. These results implicated that IL-32γ may interact with PKC isoforms and suppress inflammatory responses as an intracellular molecule. The receptor for IL-32 is still unknown; however, previous study showed that IL-32 binds to FAK, Paxillin, αVβ3 and αVβ6 integrins [75]. These studies showed that IL-32γ may interact with cytoplasmic membrane molecules to suppress inflammatory responses. Moreover, TPA-induced hyperplasia and inflammation as well as inflammatory cell infiltration were prevented in IL-32γ mice. We also found that tissues from skin tumor patients showed lower levels of IL-32 compared to healthy controls. Thus, our results demonstrated that IL-32γ inhibited skin carcinogenesis by the downregulation of cancer stemness through a reduced inflammatory microenvironment.

In GWAS analysis, we found that ITGAV and TIMP-1 were significantly associated with IL-32γ-inhibited skin tumor development. ITGAV was involved in cancer progression and was expressed in many cancer types [76–78]. In mouse skin, ITGAV cooperated with p53 to transiently promote initial skin cancer development, but ultimately resulted in decreased tumor growth [33]. ITGAV knockout mice promoted skin cancer development and ITGAV expression was positively associated with cancer cell stemness and tumorigenesis in prostate and bladder cancer [34–37]. Integrin αVβ3 increased and maintained macrophage-induced inflammatory responses [79]. ITGAV also played critical roles in the inflammatory process of a number of diseases such as cancer, atherosclerosis, or rheumatoid arthritis [80–82]. These facts correlated with our results revealing that IL-32γ suppressed cancer stemness through the inhibition of ITGAV and TIMP-1 in A431 and SK-Mel-28 skin cancer cells. Additionally, knockdown of ITGAV suppressed cancer stemness with decreased expression of cancer stem cell markers. Furthermore, expression of ITGAV was downregulated in DMBA/TPA-induced skin tissues of IL-32γ mice. Tissue microarray data showed that ITGAV expression was elevated in skin cancer tissues from patients. These data indicated that the downregulation of the anti-stemness and anti-inflammatory effects of ITGAV could be associated with the reducing effect of IL-32γ on skin carcinogenesis. TIMP-1 is a glycoprotein and plays a role in extracellular matrix composition, wound healing, and pregnancy by regulating matrix metalloproteinases. Previous studies revealed that TIMP-1 inhibited a disintegrin and metalloprotease (ADAM)-10 activity, which regulates cancer stem-like cells and tumor growth through activation of Notch signaling in colon cancer [83, 84]. However, TIMP-1 was involved in the development of various cancer, such as melanoma, colon, and acute myeloid leukemia, through exerting an inflammatory network in the tumor microenvironment [39, 40, 85]. Melanoma cells overexpressing TIMP-1 had increased anchorage-independent growth and in vivo cancer progression [40, 86]. Increased serum level of TIMP-1 was correlated with an unfavorable prognosis in patient with advanced stage melanoma [87]. In this study, we found that IL-32γ inhibited cancer sphere formation and its effect was associated with the downregulation of TIMP-1 expression in skin cancer cells. We also found that inhibition of TIMP-1 showed reduced cancer sphere formation and expression of CSC markers. The expression of TIMP-1 was also reduced in DMBA/TPA-induced skin tissues of IL-32γ mice. These data indicated that anti-inflammatory and anti-stemness effects by down-regulation of TIMP-1 could contribute to the inhibitory effect of IL-32γ.

NF-κB has been known to act as an important transcription factor for inflammation and tumor development. In CSCs, NF-κB is the key player for driving self-renewal and regulating stem cell-related genes [51]. We found that cancer cell sphere formation was reduced accompanied by decreased nuclear translocation of p50 and p65 in IL-32γ-overexpressing CSCs. In addition, inactivation of NF-κB signaling was found in DMBA-TPA-induced skin tissues from IL-32γ mice. These results implicated that IL-32γ may directly inhibit enzyme activity of IκB kinase (IKK), which was necessary for IκB ubiquitination. Supporting our results, according to a previous study from our lab, acetylation of p65 and expression p300 were inhibited in IL-32γ-overexpressing colon cancer cells and tumor tissues [10]. The combination of IL-32γ and NF-κB inhibitor, BAY, synergistically reduced TPA-induced epidermal hyperplasia, inflammation, and skin cancer sphere formation by further suppressing ITGAV and TIMP-1. Supporting our results, TPA-induced epidermal hyperplasia and inflammatory responses were attenuated in epidermal p65 knockout mice [43]. In addition, NF-κB inhibition suppressed breast CSC formation [88]. Moreover, NF-κB inhibitor treatment reduced the expression of CSC marker CD133 [89]. A previous study revealed that knockdown of TIMP-1 suppressed cell proliferation and cancer development by the inhibition of NF-κB signaling in breast cancer [49]. TIMP-1overexpressing melanoma showed increased CSC formation [40]. It has been implicated that TIMP-1 inhibition may suppress cancer stemness via inactivation of NF-κB. In hepatocellular carcinoma, the integrin αVβ3-NF-κB-HIF-1α pathway contributed to promoting a CSC phenotype [90]. It is also noteworthy that inhibition of ITGAV reduced prostate and bladder cancer stemness and these results may be related with integrin-mediated NF-κB signaling [91]. These findings suggested that the inactivation of NF-κB could contribute to IL-32γ-induced tumor growth inhibition through blockade of skin inflammation and cancer cell sphere formation with the downregulation of ITGAV and TIMP-1.

## Conclusions

This study demonstrated the inhibitory effect of IL-32γ on skin tumor development by the downregulation of ITGAV and TIMP-1 via the NF-κB signaling. Thus, IL-32γ could be a useful therapeutic molecule for skin cancer.

## Additional files

Additional file 1: **Figure S1.** IL-32 related disease network. Gene (IL-32)–disease network was analyzed based on the GWAS/OMIM/DEG records (*p* < 10^− 6^). (TIF 754 kb)

Additional file 2: **Figure S2.** Gene network analysis. A and B, The gene map of IL-32 is shown based on known functional association networks. (TIF 517 kb)

Additional file 3: **Table S1.** List and sequences of qPCR primers for mRNA expression. (TIF 98 kb)

Additional file 4: **Figure S3.** Effect of IL-32γ on tumor development. DMBA/TPA-induced skin tissues from WT and IL-32γ mice were lysed and analyzed by western blotting for PCNA, CDK4, cyclin D1, MMP-2, MMP-9, COX-2, iNOS, p-JNK, JNK, p-ERK, ERK, p-p38, p38, p-STAT3 and STAT3. β-actin was used as a loading control. (TIF 206 kb)

Additional file 5: **Figure S4.** Effect of IL-32γ on cell proliferation. HaCaT, A431 and SK-Mel-28 cells were seeded on 96-well plates (1 × 10^3^ cells per well). Cells were transfected with control or IL-32γ vector for 24 h. Cell viability was determined by MTT assay at various time points. (TIF 129 kb)

Additional file 6: **Figure S5.** Inhibition of NF-κB activity suppressed TPA-induced skin inflammation. WT and IL-32γ mice were administrated with BAY and TPA application and then sacrificed after 24 h. A, Real-time PCR analysis of different inflammatory mediators, TNF-α, IL-1β and IL-10, on mRNA isolated from skin tissue extracts. B, Real-time PCR analysis of mRNA expression of inflammatory cell markers, Ly6G, CD11b and F4/80. *n = 3*. *p < 0.05; #*p < 0.05*. (TIF 146 kb)

## Abbreviations

CSC: Cancer stem cell
DMBA: 7,12-Dimethylbenz[a]anthracene
IL: Interleukin
ITGAV: Integrin αV
NF-κB: Nuclear transcription factor-κB
TIMP-1: Tissue inhibitor of metalloproteinase-1
TPA: 12-O-Tetradecanoylphorbol-13-acetate
