# Supplementary figures and images for: Inhibition of skin carcinogenesis by suppression of NF-κB dependent ITGAV and TIMP-1 expression in IL-32γ overexpressed condition
Source: J Exp Clin Cancer Res. 2018 Nov 28;37:293. doi: 10.1186/s13046-018-0943-8 (PMC6263970; doi:10.1186/s13046-018-0943-8)

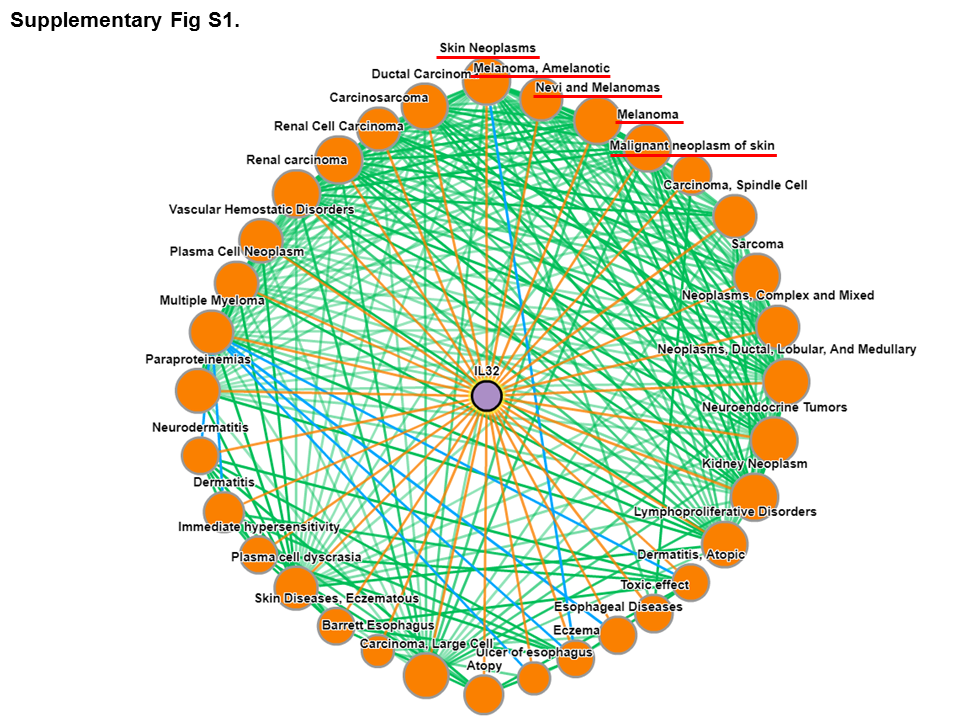

Supplement: Supplementary file 1 — Figure S1. IL-32 related disease network. Gene (IL-32)–disease network was analyzed based on the GWAS/OMIM/DEG records (p < 10− 6). (TIF 754 kb) [file 13046_2018_943_MOESM1_ESM.tif]

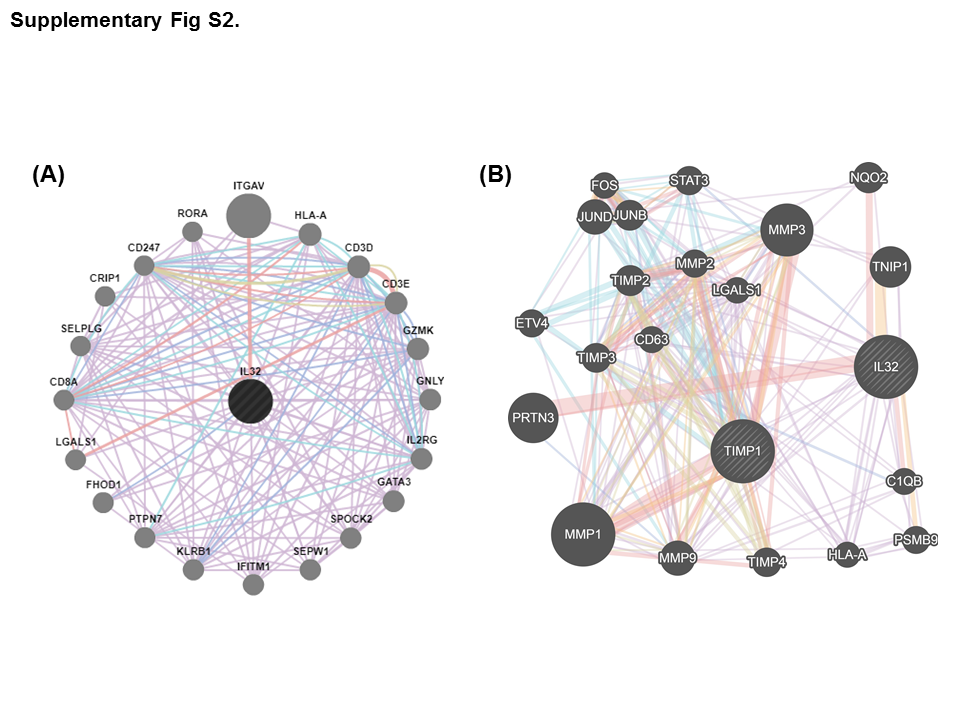

Supplement: Supplementary file 2 — Figure S2. Gene network analysis. A and B, The gene map of IL-32 is shown based on known functional association networks. (TIF 517 kb) [file 13046_2018_943_MOESM2_ESM.tif]

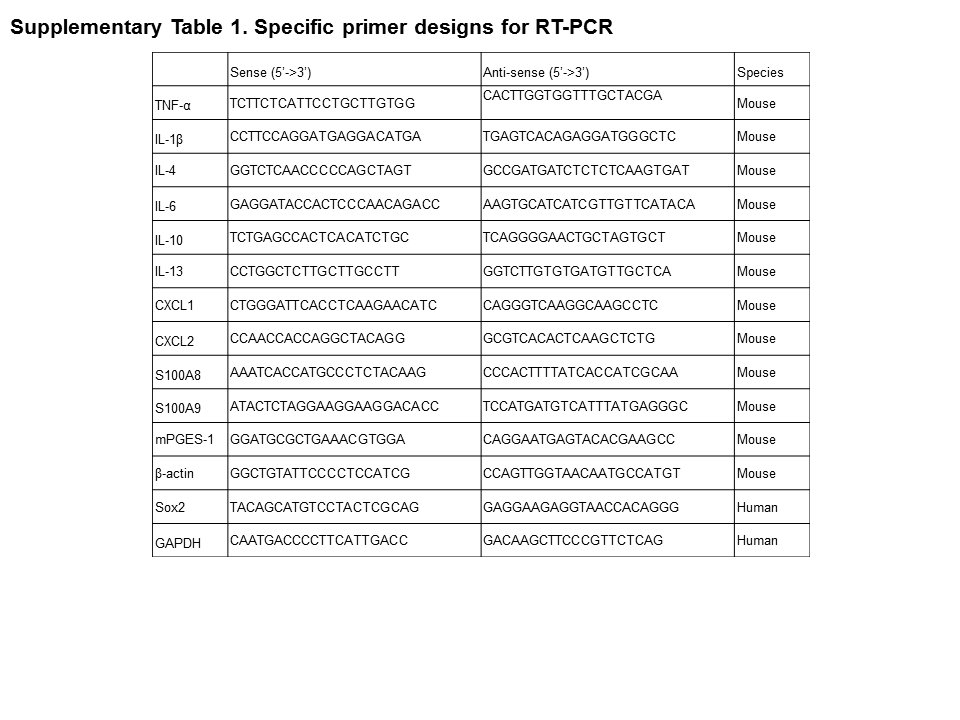

Supplement: Supplementary file 3 — Table S1. List and sequences of qPCR primers for mRNA expression. (TIF 98 kb) [file 13046_2018_943_MOESM3_ESM.tif]

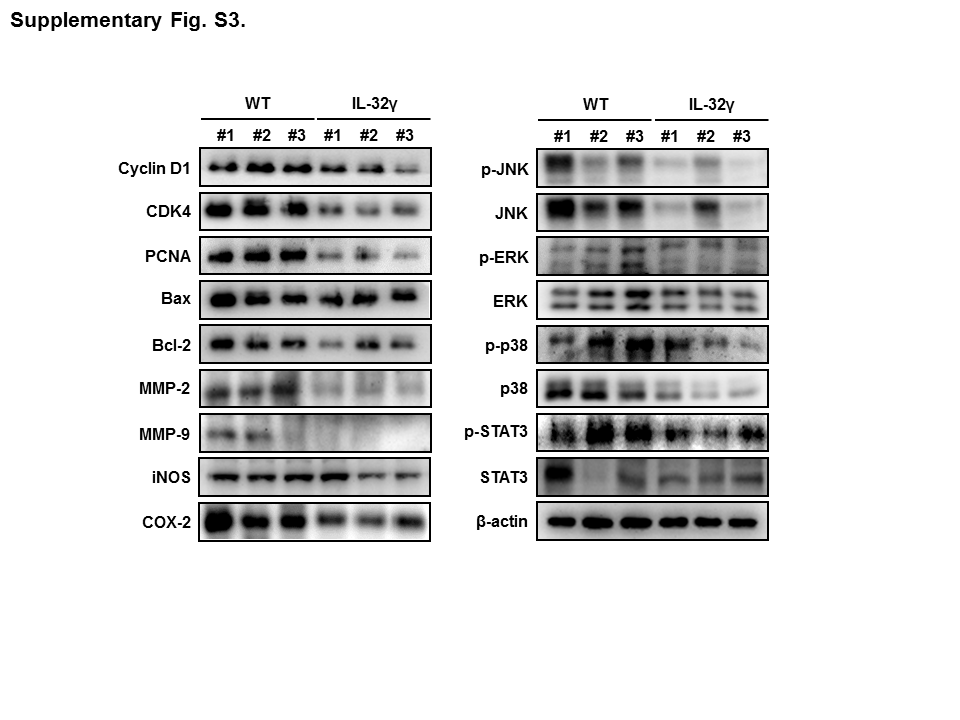

Supplement: Supplementary file 4 — Figure S3. Effect of IL-32γ on tumor development. DMBA/TPA-induced skin tissues from WT and IL-32γ mice were lysed and analyzed by western blotting for PCNA, CDK4, cyclin D1, MMP-2, MMP-9, COX-2, iNOS, p-JNK, JNK, p-ERK, ERK, p-p38, p38, p-STAT3 and STAT3. β-actin was used as a loading control. (TIF 206 kb) [file 13046_2018_943_MOESM4_ESM.tif]

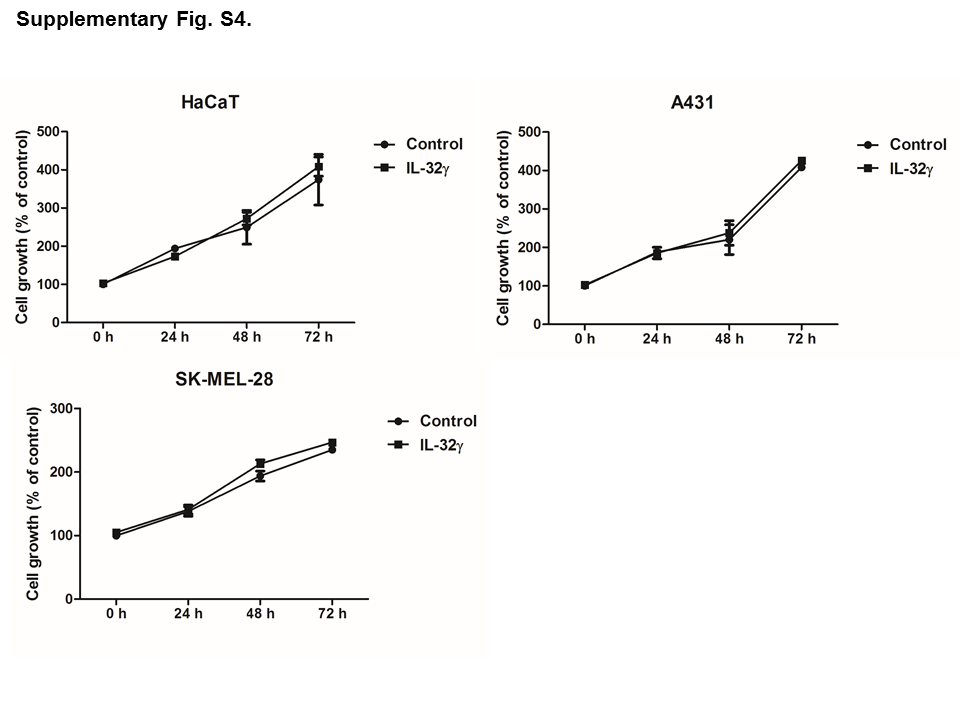

Supplement: Supplementary file 5 — Figure S4. Effect of IL-32γ on cell proliferation. HaCaT, A431 and SK-Mel-28 cells were seeded on 96-well plates (1 × 10^3^ cells per well). Cells were transfected with control or IL-32γ vector for 24 h. Cell viability was determined by MTT assay at various time points. (TIF 129 kb) [file 13046_2018_943_MOESM5_ESM.tif]

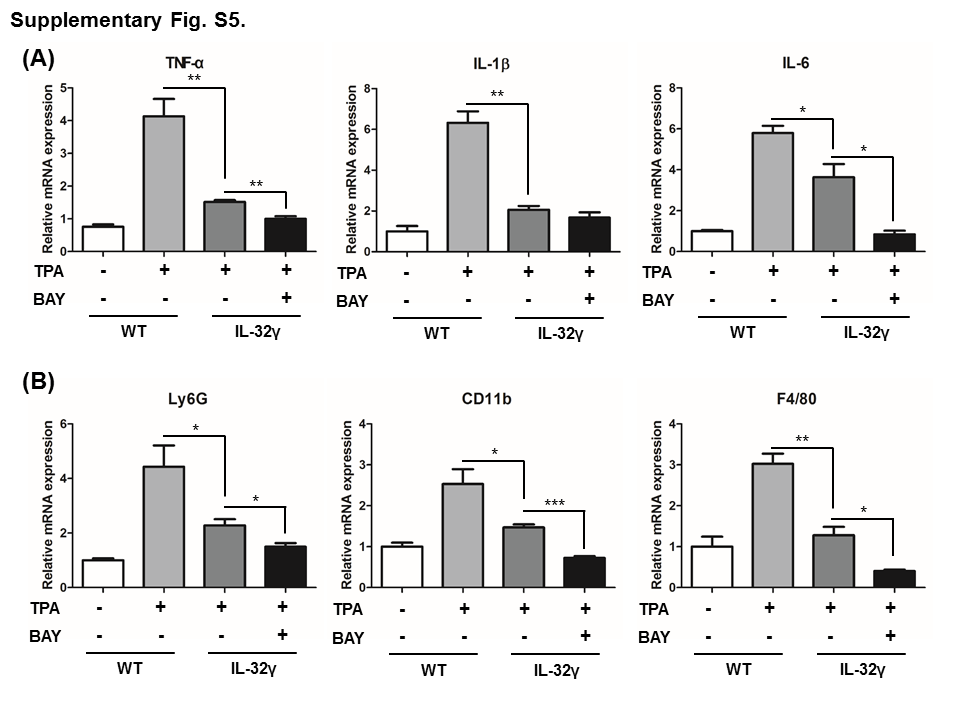

Supplement: Supplementary file 6 — Figure S5. Inhibition of NF-κB activity suppressed TPA-induced skin inflammation. WT and IL-32γ mice were administrated with BAY and TPA application and then sacrificed after 24 h. A, Real-time PCR analysis of different inflammatory mediators, TNF-α, IL-1β and IL-10, on mRNA isolated from skin tissue extracts. B, Real-time PCR analysis of mRNA expression of inflammatory cell markers, Ly6G, CD11b and F4/80. n = 3. *p < 0.05; #p < 0.05. (TIF 146 kb) [file 13046_2018_943_MOESM6_ESM.tif]
